# Machine Learning and Multimodal Biomarker Discovery in Alzheimer’s Disease

**DOI:** 10.3390/brainsci16090969

**Published:** 2026-09-14

**Authors:** Tariq Tayebi, Monique A. David, Mourad Tayebi

**Affiliations:** 1 Normanhurst Boys High School, Normanhurst, NSW 2076, Australia; 2 School of Medicine, Blacktown/Mt Druitt Clinical School, Western Sydney University, Blacktown, NSW 2148, Australia

**Keywords:** dementia, Alzheimer’s disease, diagnosis, biomarkers, machine learning

## Abstract

**Highlights:**

**What are the main findings?**

- Machine learning improves Alzheimer’s disease detection by integrating blood, cerebrospinal fluid, imaging, genetic, transcriptomic, digital, sleep, and microbiome biomarkers.
- Multimodal and interpretable models generally outperform single-biomarker approaches and can support early diagnosis, disease staging, biomarker prediction, and patient stratification.

**What are the implications of the main findings?**

- AI-assisted multimodal platforms could enable more accessible, minimally invasive, and personalized Alzheimer’s disease screening and clinical decision-making.
- Clinical translation will require longitudinal validation, diverse populations, standardized analytical pipelines, model interpretability, and appropriate regulatory oversight.

**Abstract:**

Background/Objectives: The accelerating integration of machine learning (ML) with molecular, imaging, and physiological data is transforming Alzheimer’s disease (AD) research. Methods & Results: Recent studies demonstrate that multimodal, AI-assisted platforms can enhance early diagnosis, predict biomarker trajectories, and identify novel therapeutic targets. This mini-review covers the evolving AD diagnostic and biomarker frameworks, current therapeutic strategies including recently approved anti-amyloid immunotherapies, and advances from contemporary studies employing ML across diverse data streams, ranging from cerebrospinal fluid (CSF) and plasma proteomics to Raman spectroscopy, neuroimaging, transcriptomics, and microbiome signatures. Conclusions: Collectively, they illustrate how artificial intelligence (AI) has shifted A biomarker discovery from univariate to network-based inference, achieving clinically relevant accuracy while emphasizing model interpretability. We discuss biological insights, translational implications, and persisting challenges related to validation, bias, and regulatory integration.

## 1. Introduction

Alzheimer’s disease remains the leading cause of dementia, yet its clinical diagnosis lags the onset of neuropathology by years. Conventional assessments such as CSF Aβ42, p-tau, t-tau, and amyloid/tau positron emission tomography (PET) are accurate but invasive or costly [1]. Consequently, there is an urgent need for scalable, non-invasive, and interpretable biomarkers. Parallel advances in omics and computational analytics have yielded unprecedented data volumes, necessitating AI-based approaches for pattern extraction and feature selection [2,3,4,5].

**Figure 1:**
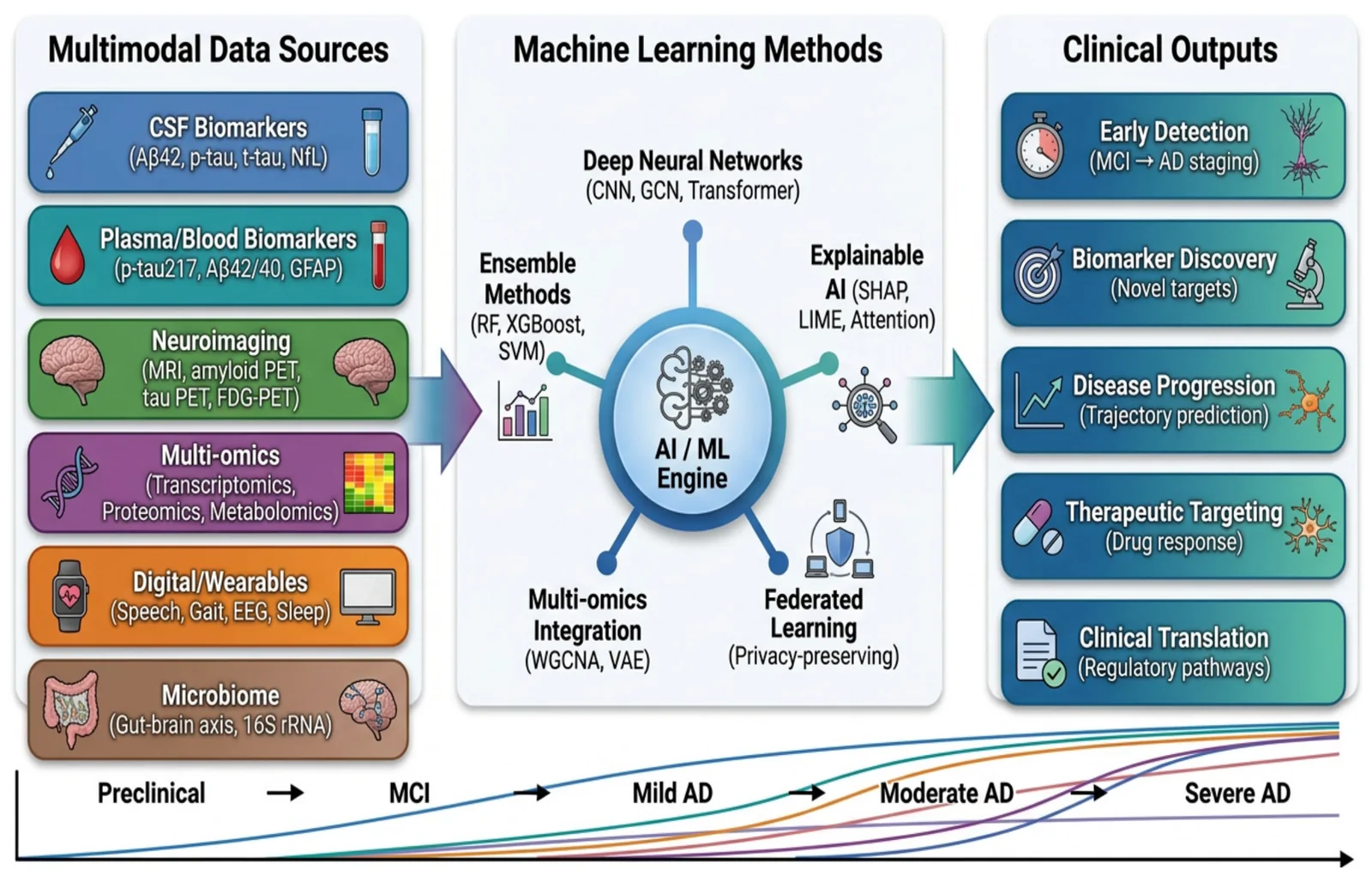
Multimodal machine learning framework for Alzheimer’s disease biomarker discovery and clinical translation. Left: diverse data modalities (genomics, plasma biomarkers, neuroimaging, clinical/cognitive assessments, gut microbiome, sleep/digital biomarkers). Center: AI/ML integration hub incorporating deep learning, explainable AI, federated learning, and multi-omics pipelines. Right: clinical outputs including AD staging, drug-target identification, clinical decision support, and trial stratification. Bottom: disease progression timeline from healthy aging through MCI to established AD. Abbreviations: CNN, convolutional neural network; GCN, graph convolutional network; RF, random forest; SVM, support vector machine; SHAP, SHapley Additive exPlanations; LIME, Local Interpretable Model-agnostic Explanations; WGCNA, weighted gene co-expression network analysis; VAE, variational autoencoder; EEG, electroencephalography; FDG-PET, fluorodeoxyglucose positron emission tomography; MCI, mild cognitive impairment; AD, Alzheimer’s disease. The color-coded progression trajectories at the base of the figure represent successive disease stages: blue, healthy/normal cognition; yellow, preclinical/subjective cognitive decline; orange, mild cognitive impairment; red, established Alzheimer’s disease dementia.

Machine learning now bridges clinical, biochemical, imaging, and digital domains, enabling multidimensional disease staging and prognosis (Figure 1). The studies reviewed herein reveal complementary innovations, from interpretable SHAP-based gene models to surface-enhanced Raman spectroscopy (SERS) immunoassays augmented by ML [6,7].

### Literature Search Strategy

A systematic search of PubMed, MEDLINE, Scopus, and Web of Science was conducted for studies published between January 2015 and March 2026. Search terms included combinations of: Alzheimer’s disease, dementia, machine learning, artificial intelligence, deep learning, biomarker, multimodal, neuroimaging, proteomics, transcriptomics, and microbiome. Inclusion criteria required peer-reviewed original research or review articles reporting ML-based analysis of AD biomarkers across at least one data modality. Preprint articles were excluded unless subsequently published in a peer-reviewed journal. Additional articles were identified through manual screening of reference lists of retrieved studies. Given heterogeneity in study populations, data modalities, and outcome definitions across included studies, direct cross-study performance comparisons are not made; instead, we describe trends and methodological advances across the literature.

## 2. Diagnostic Advances in Alzheimer’s Disease

### 2.1. From Syndromic to Biological Definitions

Historically, Alzheimer’s disease (AD) was diagnosed as a clinical syndrome based on insidious onset and progressive impairment of episodic memory and other cognitive domains, after exclusion of alternative causes [8]. The 2011 NIA–Alzheimer’s Association (NIA–AA) criteria formalized this approach across the continuum from preclinical AD to mild cognitive impairment (MCI) due to AD and dementia and explicitly introduced CSF and imaging biomarkers as supportive tools rather than core diagnostic requirements [9,10,11].

A major conceptual shift came with the 2018 NIA–AA research framework, which proposed that AD should be defined biologically by the presence of β-amyloid (A), tau (T), and neurodegeneration (N) biomarkers—the AT(N) system rather than by its clinical consequences. This harmonized in vivo diagnostics with post-mortem neuropathological definitions and facilitated standardized staging across cohorts [12,13].

In 2024, updated Alzheimer’s Association diagnostic and staging recommendations extended this biomarker-based framework into clinical practice, distinguishing “Core 1” biomarkers (primarily amyloid and tau measures) that are sufficient to establish or exclude AD pathology from “Core 2” biomarkers (e.g., NfL, GFAP, tau PET) that refine prognosis and stage.

### 2.2. CSF and Imaging Biomarkers: Implementing the AT(N) Framework

CSF biomarkers remain a cornerstone of the biological diagnosis of Alzheimer’s disease. Reduced CSF Aβ42 or the Aβ42/40 ratio reflects cerebral amyloid deposition [14,15]; elevated CSF phosphorylated tau (e.g., p-tau181, p-tau217) indicates tau pathology and has high specificity for AD [16,17,18]; and increased total tau or neurofilament light chain (NfL) reflects neuronal injury and neurodegeneration [19,20]. Overall, this CSF AT(N) profile shows strong concordance with amyloid and tau PET and predicts progression from MCI to AD dementia.

Neuroimaging provides complementary structural and functional information. Structural MRI typically reveals early hippocampal and medial temporal lobe atrophy, with later parietal and posterior cingulate involvement [21]. FDG-PET demonstrates temporo-parietal and posterior cingulate hypometabolism that helps distinguish AD from frontotemporal dementia and other conditions [22,23]. Amyloid PET with tracers such as *PIB*, *Pittsburgh*, *florbetapir*, *florbetaben*, or *flutemetamol* shows high sensitivity and specificity for cortical amyloid plaques and has been validated against autopsy [24]. Tau PET tracers (e.g., flortaucipir, MK-6240, RO948) map neurofibrillary tangle distribution and correlate closely with clinical severity, cognitive decline, and Braak stage [25,26].

Within the AT(N) framework, abnormal amyloid and tau biomarkers, whether derived from CSF or PET, are sufficient to define biologically confirmed AD, with neurodegeneration markers used for staging and prognosis [13].

### 2.3. Blood-Based Biomarkers: Towards Scalable Biological Diagnosis

The most transformative diagnostic development in the last decade has been the emergence of blood-based biomarkers (BBMs). Ultra-sensitive technologies such as SIMOA and electrochemiluminescence have enabled reliable measurement of low-abundance brain-derived proteins in plasma, facilitating non-invasive, scalable AD biomarker testing at population scale.

Across multiple large cohorts, plasma p-tau species, especially p-tau217, show diagnostic accuracies that rival CSF and PET:

- Palmqvist et al. (2020) demonstrated that plasma p-tau217 discriminated AD from other neurodegenerative diseases with AUCs ~ 0.96–0.98 and outperformed p-tau181, NfL and MRI measures [27].
- Mattsson-Carlgren et al. (2020) showed that longitudinal plasma p-tau217 increases early in the disease course and tracks tau PET and cognitive decline [28].
- Fully automated p-tau217 immunoassays now show accuracies comparable to CSF biomarkers for detecting abnormal amyloid and tau pathology [29].

Plasma Aβ42/40 ratio improves classification of amyloid PET status, especially when combined with p-tau217. Plasma NfL reflects neuroaxonal injury and predicts clinical progression across the AD continuum [30,31], while plasma GFAP is a sensitive marker of astroglial activation and early amyloid positivity.

Meta-analyses and head-to-head comparisons consistently identify p-tau217 as the single best-performing plasma biomarker for AD pathology, with Aβ42/40, NfL and GFAP providing complementary information on amyloidosis, neurodegeneration and gliosis [32].

Importantly, BBMs are now being integrated into clinical workflows as first-line, minimally invasive tests to identify individuals who require confirmatory CSF/PET testing and to monitor disease progression and treatment response. Regulatory clearance of p-tau-based plasma tests in 2025 underscores the imminent translation of BBMs into routine practice.

### 2.4. Digital and AI-Enabled Diagnostics

Digital technologies offer low-burden, high-frequency measurement of cognition and function. Speech and language analysis using machine learning can distinguish AD and MCI from normal aging with accuracies often exceeding 80–85% using acoustic and lexical features [33]. Smartphone-based tasks and passive monitoring of typing, navigation and app usage capture subtle executive and memory changes not readily detected in clinic-based assessments [34].

Wearables and ambient sensors measure gait, sleep, physical activity and social interaction. Longitudinal gait speed and variability, circadian rhythm disruption, and activity patterns have all been associated with incident MCI and dementia [35].

## 3. Therapeutic Strategies in Alzheimer’s Disease

### 3.1. Symptomatic Pharmacological Therapies

Despite the rise of disease-modifying therapies (DMTs), symptomatic treatments remain foundational. Three acetylcholinesterase inhibitors (AChEIs): donepezil, rivastigmine, and galantamine are approved for mild-to-moderate AD in many jurisdictions. Meta-analyses show modest but significant improvements or slower decline in cognition, global function and activities of daily living [36].

Memantine, an NMDA-receptor antagonist, is indicated for moderate-to-severe AD and can be used alone or in combination with an AChEI. Randomized trials and meta-analyses report small benefits on cognition, behavior and function, particularly in more advanced stages [37]. Guidelines emphasize individualized use based on stage, comorbidities and side-effect profiles, with regular review and deprescribing considered when benefit is uncertain [38].

### 3.2. Non-Pharmacological and Multidomain Lifestyle Interventions

Non-pharmacological interventions are central to a holistic care model. Cognitive stimulation therapy, structured exercise, social engagement, environmental modification and caregiver education improve quality of life and may influence cognitive trajectories [39].

The Finnish Geriatric Intervention Study to Prevent Cognitive Impairment and Disability (FINGER) is the landmark multidomain trial. It showed that a 2-year intervention combining nutritional counseling, physical exercise, cognitive training and vascular risk management improved or maintained cognition in at-risk older adults compared with general health advice [40]. Follow-up analyses and WW-FINGERS initiatives, including FINGER-NL, are evaluating generalizability and long-term effects across diverse populations.

In individuals with prodromal or early AD, multidomain programs including cognitive training and physical activity, sometimes combined with medical nutrition (e.g., Fortasyn Connect), have shown benefits on memory performance and functional outcomes [41]. Although effect sizes are modest, such interventions are safe, scalable and likely synergistic with pharmacological DMTs by reducing vascular and metabolic comorbidities and enhancing cognitive reserve.

### 3.3. Disease-Modifying Anti-Amyloid Monoclonal Antibodies

After decades of negative trials, anti-amyloid monoclonal antibodies (mAbs) have provided the first robust evidence of disease modification in early AD and have received regulatory approval in multiple regions.

Lecanemab is a humanized IgG1 mAb targeting soluble Aβ protofibrils. In the phase 3 CLARITY AD trial, involving participants with MCI due to AD or mild AD dementia with biomarker-confirmed amyloidosis, lecanemab significantly reduced amyloid plaque burden and produced ~27% less decline on the CDR-SB over 18 months, with convergent benefits on key secondary outcomes. Health-related quality-of-life analyses showed smaller declines on EQ-5D-5L and QOL-AD and reduced caregiver burden, consistent with a modest but clinically meaningful slowing of progression.

ARIA-E (edema) and ARIA-H (micro/macro-hemorrhages) were the main safety concerns, particularly among APOE ε4 homozygotes, necessitating MRI-based monitoring and careful risk–benefit discussions.

Donanemab, an IgG1 mAb recognizing N-terminal pyroglutamate-modified Aβ, also showed substantial efficacy. In TRAILBLAZER-ALZ 2, donanemab significantly slowed clinical decline on the integrated Alzheimer’s Disease Rating Scale (iADRS) and CDR-SB, with approximately 35% relative slowing, especially in participants with low-to-intermediate tau burden [42]. Donanemab also produced very high rates of amyloid PET negativity, supporting a plaque-clearance mechanism. ARIA rates were again substantial, including symptomatic and rare fatal events, underscoring the need for stringent eligibility criteria, APOE genotyping, and structured MRI surveillance.

Both lecanemab and donanemab are now approved or under regulatory review in multiple jurisdictions, but implementation requires infusion capacity, biomarker infrastructure and trained multidisciplinary teams, raising major health-system and equity challenges.

### 3.4. Other Disease-Modifying Strategies

#### 3.4.1. Anti-Tau Monoclonal Antibodies and Tau-Targeted Therapies

Tau pathology is more closely associated with clinical severity than amyloid, making tau an appealing target [43]. However, several first-generation anti-tau mAbs, including gosuranemab, tilavonemab and zagotenemab, failed to meet primary endpoints in phase 2–3 AD trials despite acceptable safety profiles. To date, tau-directed antibodies have not demonstrated clinically meaningful effects in symptomatic AD, though newer agents and earlier intervention windows remain under investigation.

Other tau-targeting approaches include antisense oligonucleotides to reduce tau expression, small-molecule tau aggregation inhibitors, microtubule stabilizers, and emerging proteolysis-targeting chimera (PROTAC) strategies that selectively degrade tau [44]. Most remain in early-phase trials.

#### 3.4.2. Multi-Target and Next-Generation DMTs

Given the multifactorial nature of AD, current pipelines increasingly focus on multi-target and pathway-agnostic approaches. These include microglial and complement modulators, inflammasome inhibitors, metabolic and mitochondrial stabilizers, synaptic plasticity enhancers, and agents targeting vascular and blood–brain barrier dysfunction. Rational combinations of amyloid, tau and non-amyloid therapies are expected to be key for future disease-modifying regimens.

Emerging evidence also implicates mitochondrial homeostasis dysregulation in the pathogenesis of AD, with TDP-43, an RNA-binding protein better known from ALS and recently identified as a mediator of mitochondrial dysfunction in this setting [45]. TDP-43 pathology disrupts mitochondrial biogenesis, calcium buffering, and mitophagy, contributing to synaptic failure and neuroinflammation. These findings position mitochondrial and TDP-43 pathways as additional ML-detectable biomarker targets, particularly for stratifying patients with mixed or atypical neurodegenerative pathology.

### 3.5. Digital Therapeutics and Technology-Enabled Care

Digital therapeutics are being developed as active treatment tools rather than just measurement devices. Computerized cognitive training programs produce small-to-moderate improvements in memory and executive function in MCI and early AD [46,47]. VR and XR-based interventions can deliver immersive spatial navigation and memory tasks, and early studies suggest feasibility and positive user experience in MCI and early dementia [48].

Remote platforms supporting exercise, diet, medication adherence and caregiver education can deliver multidomain interventions at scale and with high personalization, particularly when integrated with wearable data and AI-based decision support [49]. As infusion-based DMTs become more widely used, such technology-enabled care pathways will be critical for monitoring treatment response, ARIA symptoms, functional outcomes, and caregiver burden.

Machine learning is increasingly positioned not only as a diagnostic tool but also as a key enabler of therapeutic development and monitoring in AD. ML-based patient stratification using multimodal biomarker profiles can identify individuals most likely to benefit from anti-amyloid immunotherapy, enabling precision trial enrolment and reducing the cost of biomarker-confirmed eligibility [50]. Longitudinal ML frameworks that integrate clinical, imaging, and fluid biomarker trajectories provide dynamic treatment monitoring, allowing for early detection of ARIA or disease progression that may necessitate dosing adjustments. This bidirectional relationship between AI-driven biomarker discovery and therapeutic application represents a paradigm shift towards precision medicine in AD.

## 4. Machine Learning in Fluid Biomarker Discovery

### 4.1. Blood-Based and Raman Platforms

Resmi et al. (2024) introduced a SERS-immunoassay integrated with ML to detect Aβ40, Aβ42, p-tau, and t-tau in plasma at attomolar sensitivity, distinguishing mild cognitive impairment (MCI) from AD with high specificity [7]. Random-forest and support-vector models trained on spectral signatures provided accurate classification, suggesting a low-cost alternative to CSF assays (Table 1).

Similarly, Khorsand et al. (2025) applied multiple classifiers, KNN, SVM, RF, and decision trees, to biochemical panels (neprilysin, β-secretase, urinary formic acid), achieving ~94% sensitivity [51]. These approaches underscore ML’s potential to integrate heterogeneous biochemical inputs for point-of-care screening. AlMansoori et al. (2024) further demonstrated that combining blood-based gene expression profiles with SNP data in an ensemble ML framework achieved 87% accuracy in predicting early-onset AD, underscoring the value of integrating genetic and transcriptomic inputs [52] (Table 1).

### 4.2. Systematic Trends

A 2023 systematic review by Blanco et al. (2023) found increasing adoption of multimodal algorithms combining fluid biomarkers, neuroimaging, and genetics, outperforming unimodal models for predicting MCI-to-AD conversion [50]. Yet, most studies lacked longitudinal diversity and white-box interpretability, emphasizing the need for transparent frameworks.

## 5. Multimodal Integration: Imaging, Sleep, and Clinical Features

MRI and PET remain crucial, but ML enables surrogate prediction. Jasodanand et al. (2025) trained multimodal neural networks across >12 000 participants to predict amyloid and tau PET status from accessible measures (MRI + cognitive + clinical data), achieving AUROC 0.79–0.84 [53]. This framework can pre-screen trial candidates for anti-amyloid therapies, cutting PET-related costs. Complementarily, Gaeta et al. (2024) combined polysomnography-derived quantitative EEG with clinical variables in a gradient-boosting model to estimate CSF Aβ42, p-tau, and t-tau concentrations, identifying sleep-architecture biomarkers as non-invasive correlates of AD pathology [54] (Table 1).

MRI-based models reviewed by Orlunwo et al. (2024) demonstrated that ensemble algorithms integrating MRI and CSF biomarkers yield earlier classification of prodromal AD than traditional volumetric measures [55]. Shah et al. (2023) and Zhou et al. (2025) extended this multimodal approach with an interpretable graph convolutional network (GCN) integrating MRI morphology, PET metabolic data, and genetic features, achieving 92.3% classification accuracy for AD and MCI with GradCAM-based region-level explanations [56,57] (Table 1).

Brain parcellation approaches further enhance the sensitivity of ML models in imaging-based AD classification. The Desikan–Killiany atlas, implemented via FreeSurfer, provides standardized cortical thickness measurements across 68 regions of interest, enabling ML models to leverage region-specific atrophy signatures beyond hippocampal volume alone. Studies employing parcellation-guided feature extraction have demonstrated superior classification accuracy compared with whole-brain volumetrics, particularly for identifying subtle cortical thinning in preclinical and prodromal AD stages [58,59]. Integrating automated parcellation pipelines with deep learning models represents a scalable approach for high-throughput screening in large-scale neuroimaging cohorts.

Multimodal integration, as employed in the studies reviewed here, refers to the computational fusion of two or more data streams such as neuroimaging, fluid biomarkers, genetic data, and clinical assessments to generate a unified predictive model. Three principal integration strategies are employed: early fusion (concatenating features before model training), late fusion (combining outputs of modality-specific models), and intermediate fusion (learning shared latent representations via multi-stream architectures). Multimodal approaches consistently outperform single-modality models in AD classification and progression prediction, because different data streams capture complementary aspects of the disease, reducing reliance on any single potentially noisy or invasive biomarker.

**Table 1:** Summary of key machine learning studies in AD multimodal biomarker research reviewed herein.

Study/Year	Population	Data Modalities	ML Algorithm(s)	Validation Strategy	Key Performance	Limitations
Resmi et al., 2024 [7]	MCI and AD patients (plasma)	Plasma SERS-immunoassay; Aβ40, Aβ42, p-tau, t-tau	Random Forest, SVM	MCI vs. AD classification	Attomolar sensitivity; high specificity vs. CSF	Single-center; specialist SERS equipment required
Khorsand et al., 2025 [51]	AD patients and controls	Biochemical panels (neprilysin, β-secretase, urinary formic acid)	KNN, SVM, Random Forest, Decision Trees	AD vs. control classification	~94% sensitivity	Limited biomarker panel; external validation lacking
AlMansoori et al., 2024 [52]	Early-onset AD cohort	Blood gene expression + SNP data	Ensemble ML	Early-onset AD classification	87% accuracy	Limited cohort size; potential overfitting risk
Jasodanand et al., 2025 [53]	>12,000 participants (multi-site)	MRI + cognitive scores + clinical features	Multimodal neural networks	Amyloid/tau PET status prediction	AUROC 0.79–0.84	PET-based labels only; no longitudinal follow-up
Gaeta et al., 2024 [54]	AD and MCI patients with polysomnography	Polysomnography-derived qEEG + clinical features	Gradient Boosting	CSF Aβ42/p-tau/t-tau biomarker estimation	Sleep-architecture biomarkers validated	Small sample; sleep data difficult to collect at scale
Orlunwo et al., 2024 [55]	Prodromal AD patients	MRI + CSF biomarkers	Ensemble algorithms	Prodromal AD vs. controls	Earlier classification vs. volumetric MRI alone	Reliance on CSF; limited to prodromal stage
Zhou et al., 2025 [57]	AD and MCI cohort	MRI morphology + PET + genetic features	Interpretable Graph Convolutional Network (GCN)	AD vs. MCI classification	92.3% classification accuracy; GradCAM explanations	GradCAM interpretation requires neuroimaging expertise
Wang et al., 2024 [6]	AD transcriptomics datasets + cell models	Transcriptomics (gene expression)	WGCNA + SHAP-interpretable ML	Cell model validation of hub genes	MYH9/RHOQ identified as hub genes	Transcriptomic only; cell model validation limited
Eteleeb et al., 2024 [60]	ROS/MAP cohort (post-mortem)	Genomics + transcriptomics + proteomics + metabolomics	Multi-omics integration and profiling	Subtype discovery in post-mortem tissue	Molecularly distinct AD subtypes identified	Post-mortem only; no ante-mortem validation
Stripelis et al., 2024 [61]	Distributed neuroimaging (multi-center)	Neuroimaging (MRI)	Federated Deep Learning (MetisFL)	Federated vs. centralized training comparison	Sub-1% accuracy loss vs. centralized training	Communication overhead; limited to neuroimaging

## 6. Omics and Interpretable Gene-Level Modeling

Transcriptomic integration offers mechanistic insight. Wang et al. (2024) combined differential expression, WGCNA, and SHAP-interpretable ML to highlight MYH9 and RHOQ as potential AD hub genes, validated experimentally in cell models [6]. This “explainable AI” paradigm enables biological validation of computational predictions that address the black-box critique (Table 1).

Winchester et al. (2023) further emphasized that both supervised and unsupervised ML models can reveal multi-scale biomarker networks spanning proteomic, microbial, and digital domains, provided that data diversity and transparent reporting improve [62].

Complementing transcriptomic models, Eteleeb et al. (2024) performed high-throughput multi-omics profiling integrating genomics, transcriptomics, proteomics, and metabolomics across post-mortem brain samples from the ROS/MAP cohort, revealing molecularly distinct AD subtypes with divergent pathway activation [60] (Table 1). Notably, one subtype exhibited immune-driven tau accumulation independent of amyloid load, suggesting that personalized therapeutic targeting based on omics-defined patient stratification may be essential for clinical trials.

A critical challenge in high-dimensional omics-based ML is the risk of overfitting, particularly when the number of features substantially exceeds sample size—a common scenario in transcriptomic and proteomic studies. Methodological safeguards including nested cross-validation, leave-one-out cross-validation (LOOCV), and spin-permutation testing for spatial autocorrelation in neuroimaging should be considered as minimum standards for performance evaluation. The use of resampling-based permutation null distributions and independent hold-out cohorts is essential to differentiate genuine signal from chance-level performance in high-dimensional feature spaces [63,64].

## 7. The Gut–Brain Axis and Microbiome-Linked Biomarkers

Alzheimer’s disease is increasingly recognized as a systemic condition, with peripheral networks including the gastrointestinal tract playing a bidirectional role in neurodegeneration. This perspective has expanded the biomarker landscape to include microbial and metabolic signatures detectable beyond the brain.

Beyond classical biomarkers, Loh et al. (2024) detailed how gut-microbiota dysbiosis precedes neurodegenerative changes, proposing probiotics, postbiotics, and dietary modulation as adjunctive strategies [65]. Integrating microbial metabolites into ML frameworks could identify microbial–host signatures predictive of early AD or depression comorbidity, an area aligned with emerging probiotic intervention trials.

## 8. Challenges and Future Directions

Despite remarkable progress, key barriers remain:

- Standardization and reproducibility: Divergent preprocessing pipelines impede cross-study comparability. Variability in the process of feature extraction can lead to model instability and inflated performance estimates. For instance, many classifiers such as Random Forest and Support Vector Machines provide different feature importance rankings depending on how input features are scaled. A promising mathematical framework for harmonizing heterogeneous multi-center data streams is multiset canonical correlation analysis (mCCA), which identifies shared variance components across multiple datasets while preserving site-specific effects. Embedding mCCA-based harmonization layers into federated learning pipelines may reduce batch effects without compromising data privacy. In the context of AD, cascaded multi-view CCA (CaMCCo) has been applied to fuse clinical, neuroimaging, proteomic, and genomic features, achieving superior multi-class diagnostic performance over single-modality approaches [66].
- Data diversity: Most training datasets over-represent European ancestry, limiting generalizability. This imbalance leads to biased predictions and misidentification and interpretation of biomarkers. In AD, over-representation in datasets leads to inconsistent performance, particularly in small-scale ML programs or research looking to derive broadly applicable conclusions. Out-of-distribution (OOD) generalization—the ability of a model trained on one cohort to perform reliably on data from different centers, ethnicities, or acquisition protocols—represents a critical but underexplored challenge. Strategies such as domain adaptation, adversarial training, and cohort-stratified validation are essential for ensuring genetic and phenotypic portability across diverse populations [67].
- Interpretability: Adoption of SHAP, LIME, and counterfactual explanations must accompany performance metrics to gain clinician trust. ML models must be interpretable to clinicians and researchers to understand certain predictions and to validate results with traditional non-ML software and biomarker research. Features like SHAP and LIME attribute importance to input features, providing essential explanations.
- Clinical translation: Regulatory validation and cost–benefit analyses are needed before integration into primary care or digital diagnostics. While achieving performance in research environments, many widely used ML models and tools lack standardized results and reports for clinical practice. This method may also not align with traditional regulations such as FDA approval for medical devices, which require rigorous demonstration of safety, efficacy, reproducibility, and clinical validation. Traditional frameworks also emphasize Good Clinical Practice (GCP) and post-market surveillance; requirements are often not met by rapidly evolving AI/ML tools that undergo continuous learning and model updates. Further research is required to assess the benefit of AI-assisted biomarker analysis in the medical field, while widespread adoption remains limited due to inaccessibility and the infancy of ML programs and hardware.

The development of ML in relation to AD has accelerated significantly over the past decade, allowing the production of various emerging solutions. Federated learning is an ML technique gaining traction as a tool that could efficiently train models across multiple decentralized servers, leaning to preserving privacy in a locally trained and iterative process; for example, Stripelis et al. (2024) demonstrated with MetisFL that federated deep learning across distributed neuroimaging repositories converges at sub-1% accuracy loss compared with centralized training [61] (Table 1). Synthetic data augmentation uses AI and ML programs that create new artificial data points that expand a dataset and can improve a model. By modifying the existing data and creating new data, this relatively new tool may improve accuracy in small or medium datasets, or over-specific data. Another emerging solution are hybrid models that integrate traditional biomarkers with data-driven machine learning structures. These models embed domain knowledge into the structure of neural networks and ML models. These solutions tackle the underdeveloped infrastructure surrounding ML practicality in AD research.

## 9. Conclusions

Collectively, these studies signify a paradigm shift from single-modality biomarkers to software-enabled multimodal ecosystems that integrate biochemical, imaging, electrophysiological, and microbial data. The convergence of interpretability, ultra-sensitivity, and accessibility is bringing precision diagnostics utilizing both medical analysis and ML algorithms closer to clinical reality. Future research should emphasize longitudinal, population-diverse datasets, integration with wearable and microbiome analytics, and translation into cost-effective screening tools that can be analyzed and implemented in a widespread, beneficial fashion, across clinics, practices, and research centers.

## Data Availability

No new data were created or analyzed in this study. Data sharing is not applicable to this article.
